# The interplay between ferroptosis and inflammation: therapeutic implications for cerebral ischemia-reperfusion

**DOI:** 10.3389/fimmu.2024.1482386

**Published:** 2024-11-08

**Authors:** Yuxuan He, Jingyi Wang, Chunmiao Ying, Kang Li Xu, Jingwen Luo, Baiqiao Wang, Jing Gao, Zaitian Yin, Yunke Zhang

**Affiliations:** ^1^ The First Clinical Medical College of Henan University of Chinese Medicine, Zhengzhou, Henan, China; ^2^ Faculty of Chinese Medicine of Macau University of Science and Technology, Macao, Macao SAR, China

**Keywords:** ferroptosis, inflammation, cerebral ischemia-reperfusion, therapeutic strategy, pharmacology

## Abstract

Stroke ranks as the second most significant contributor to mortality worldwide and is a major factor in disability. Ischemic strokes account for 71% of all stroke incidences globally. The foremost approach to treating ischemic stroke prioritizes quick reperfusion, involving methods such as intravenous thrombolysis and endovascular thrombectomy. These techniques can reduce disability but necessitate immediate intervention. After cerebral ischemia, inflammation rapidly arises in the vascular system, producing pro-inflammatory signals that activate immune cells, which in turn worsen neuronal injury. Following reperfusion, an overload of intracellular iron triggers the Fenton reaction, resulting in an excess of free radicals that cause lipid peroxidation and damage to cellular membranes, ultimately leading to ferroptosis. The relationship between inflammation and ferroptosis is increasingly recognized as vital in the process of cerebral ischemia-reperfusion (I/R). Inflammatory processes disturb iron balance and encourage lipid peroxidation (LPO) through neuroglial cells, while also reducing the activity of antioxidant systems, contributing to ferroptosis. Furthermore, the lipid peroxidation products generated during ferroptosis, along with damage-associated molecular patterns (DAMPs) released from ruptured cell membranes, can incite inflammation. Given the complex relationship between ferroptosis and inflammation, investigating their interaction in brain I/R is crucial for understanding disease development and creating innovative therapeutic options. Consequently, this article will provide a comprehensive introduction of the mechanisms linking ferroptosis and neuroinflammation, as well as evaluate potential treatment modalities, with the goal of presenting various insights for alleviating brain I/R injury and exploring new therapeutic avenues.

## Graphical Abstract

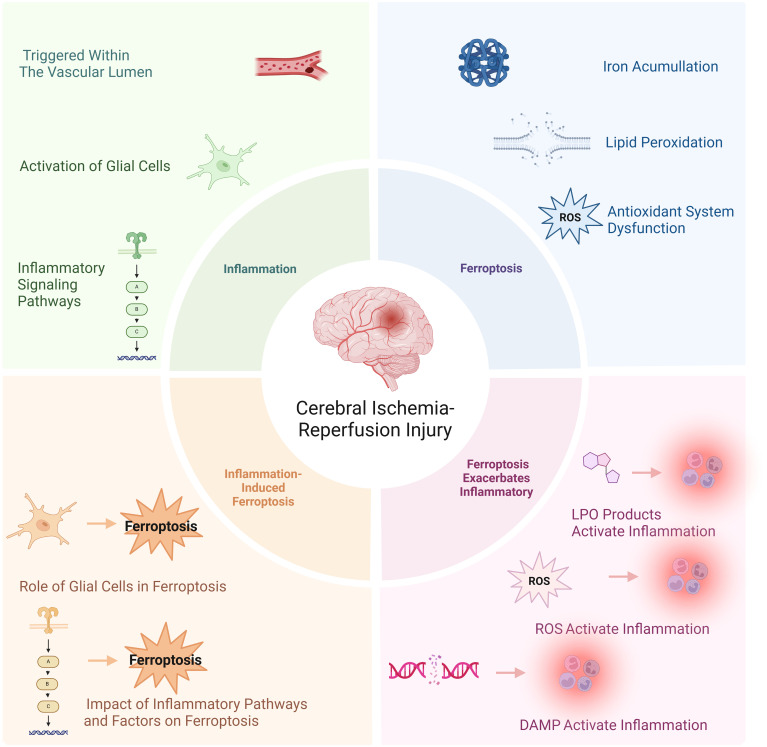

## 1 Introduction

Stroke remains a significant global health issue, marked by alarmingly high rates of both incidence and mortality. In the year 2019, it was responsible for approximately 6.55 million fatalities and impacted 143 million individuals, imposing a heavy burden on both society and those affected (1). Ischemic strokes comprise 71% of all stroke occurrences, typically resulting from the narrowing or blockage of cerebral arteries (2, 3). This situation leads to insufficient local blood supply, pronounced hypoxia, and disturbances in energy metabolism. As a result, these factors initiate oxidative stress and cell death, contributing to the injury of brain tissue (4). Despite the brain representing just about 2% of the total body weight, it utilizes roughly 20% of the oxygen and glucose in the body, underscoring the vital role of cerebral blood circulation in providing necessary nutrients to the brain (5). Inadequate blood flow to the brain can result in neuron death within five minutes after circulation stops. Prompt reperfusion can alleviate the harmful effects associated with cerebral ischemia, with thrombolytic therapies and mechanical thrombectomy being the most commonly utilized methods (6). Alteplase, a tissue plasminogen activator (tPA), is recognized globally as the primary thrombolytic agent (7, 8). Nevertheless, the process of reperfusion itself may inflict additional damage on ischemic brain tissues, a situation referred to as cerebral I/R injury (9).

Although our grasp of the exact mechanisms behind cerebral I/R remains incomplete, increasing evidence indicates that ferroptosis significantly contributes to this phenomenon (10). Ferroptosis represents a unique type of programmed cell death, distinguishing itself from apoptosis and necrosis. It is marked by an abnormal buildup of iron, which leads to an excessive generation of free radicals. This excessive radical production ultimately causes LPO of the cell membrane, resulting in cell death (11). Recent research has shed light on the intricate relationships between ferroptosis and cerebral I/R, encompassing LPO, damage to cell membranes, disruption of the antioxidant defense system, and inflammation—all of which are factors that contribute to cell death and worsen damage to brain tissue (12). Current strategies designed to alleviate ferroptosis induced by cerebral I/R, including the use of antioxidants and iron chelators (13, 14), show limited success. This limitation highlights the critical need for new therapeutic approaches. Inflammation also acts as a driving force in cerebral I/R, usually appearing as a reaction to tissue damage. Cellular injury from ischemic hypoxia instigates an inflammatory response; injured neurons release pro-inflammatory cytokines and chemokines that draw immune cells, such as microglia and neutrophils, to the injury site (15, 16). These immune cells generate further inflammatory mediators, intensifying the local inflammatory response and potentially causing additional neuronal damage (17).

Although ferroptosis and inflammation are recognized as separate injury pathways, recent studies are increasingly highlighting the significant interactions that exist between them (18, 19). Exploring these mechanisms not only deepens our comprehension of cerebral I/R but also lays the groundwork for novel approaches to disease modification and the development of treatment strategies. This article succinctly outlines the crosstalk mechanisms between inflammation and ferroptosis and suggests potential therapeutic strategies, including pharmaceutical interventions, enriched environments, and the use of exosomes. On this basis, we delve deeper into potential therapeutic mechanisms for managing both inflammation and ferroptosis. Grounded in established theoretical frameworks and supported by the literature, these strategies showcase promising applications. They aim to offer insights for the treatment of cerebral I/R-related ferroptosis and inflammation.

## 2 Ferroptosis and cerebral I/R

**Figure 1:**
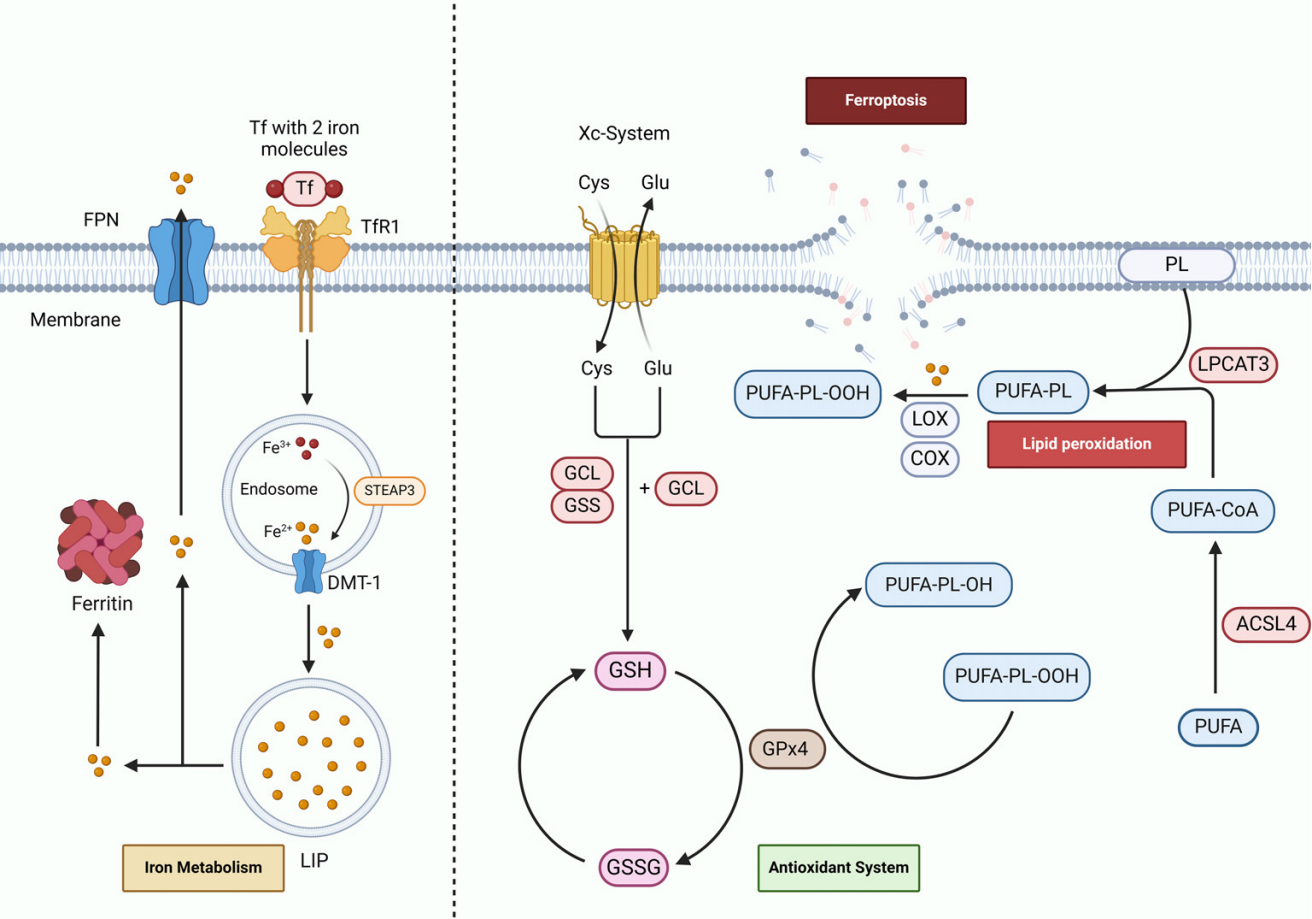
Mechanisms of ferroptosis in cerebral I/R. Tf binds to Fe3^+^ and is internalized into the cell via TfR1 through endocytosis. Within the cell, Fe3^+^ is reduced to Fe2+ by STEAP3 and subsequently stored in the labile iron pool (LIP) and ferritin via DMT-1. Dysregulation of iron metabolism can result in iron accumulation and ferroptosis. Iron within cells is exported extracellularly through FPN. ACSL4 and LPCAT3 incorporate free polyunsaturated fatty acids (PUFAs) into phospholipids, forming PUFA-PL. Lipoxygenases (LOX), cyclooxygenases (COX), and Fe2+ catalyze the oxidation of PUFA-PL into PUFA-PL hydroperoxides (PUFA-PL-OOH), which triggers lipid peroxidation (LPO). The antioxidant system, via the Xc-System, synthesizes GSH from amino acids. GPx4 subsequently reduces PUFA-PL-OOH to PL-OH, thereby mitigating LPO.

In the year 2012, Stockwell and his research team made a significant breakthrough in the field of cell biology by identifying a novel form of cell death, termed ferroptosis, which is induced by erastin and is not dependent on apoptosis (20). The uncovering of ferroptosis has spurred a considerable amount of research aimed at unraveling its underlying mechanisms and exploring the broader implications of this unique cell death pathway. Ferroptosis stands apart from the more commonly understood mechanisms of cell death, such as apoptosis and necrosis, due to its distinct characteristics. The defining feature of ferroptosis is the presence of iron overload within the cellular environment, which consequently leads to the peroxidation of lipids. This process is intricately regulated by various factors that include iron metabolism, lipid metabolism, and the maintenance of redox homeostasis (21). This multifaceted interaction highlights the complexity of ferroptosis and showcases its pivotal role in cellular physiology, as illustrated in **Figure 1**.

### 2.1 Abnormal iron metabolism

Iron is crucial for numerous biological functions in the brain, playing key roles in processes like mitochondrial respiration, myelin formation, neurotransmitter production, and other cellular functions (22). Disruptions in iron homeostasis, leading to iron accumulation, can compromise these functions. In the context of cerebral I/R, ischemia causes the release and accumulation of iron in neurons and glial cells (23). Subsequent reperfusion, which reestablishes blood flow, induces oxidative stress and inflammatory responses, thereby exacerbating iron accumulation. Excess iron can generate highly reactive hydroxyl radicals via the Fenton reaction. These radicals attack the lipid components of cell membranes, intensifying LPO and ultimately leading to ferroptosis (24, 25). In response to hypoxic injury resulting from cerebral ischemia, levels of hypoxia-inducible factor 1-alpha (HIF-1α) significantly rise, which increases iron uptake through TfR1 (26). Research has shown that patients who have suffered a stroke demonstrate markedly higher serum levels of iron and hepcidin, leading to decreased expression of FPN, reduced iron export (27), and subsequent build-up of iron, thereby creating conditions conducive to ferroptosis (28). Additionally, the compromised integrity of the blood-brain barrier (BBB) allows a greater influx of circulating iron into the brain tissue (29). In the transient middle cerebral artery occlusion (tMCAO) model, following oxygen-glucose deprivation (OGD), transferrin leaks across the BBB, and after ischemic events, DMT-1 levels in the affected brain region double, causing an accumulation in neighboring neurons (30).

### 2.2 LPO

Uncontrolled LPO is a significant factor leading to the destruction of cellular membrane structures and is a primary contributor to the process known as ferroptosis (31). LPO is an oxidative process targeting polyunsaturated fatty acids (PUFAs), triggered by free radicals. PUFAs are essential for maintaining the fluidity and flexibility of cell membranes, which is vital for effective signal transmission within neural pathways (32). However, due to the presence of unstable double bonds, PUFAs are highly prone to oxidation, subsequently making them major contributors to LPO (33). During this process, lipid peroxides form through a chemical interaction among lipids, oxygen, and iron. Once these lipid peroxides are generated, they interact with Fe2^+^, resulting in the formation of peroxyl radicals. These radicals further extract hydrogen atoms from adjacent acyl chains in the membrane, thereby propagating the LPO cycle. This reaction not only undermines the structural integrity of cell membranes but also diminishes their functionality (31).

In the particular case of cerebral I/R, the overexpression of ACSL4 promotes the acylation of PUFAs such as arachidonic acid (AA) and adrenic acid (AdA), increasing their abundance in the cell membrane. This heightened presence makes the membrane more sensitive to oxidative stress, leading to LPO and exacerbating ferroptosis in ischemic brain injury. Conversely, knockout of ACSL4 provides protective effects for mice, significantly mitigating the associated brain damage caused by ischemic events (34). Enzymatic reactions also play a vital role in promoting LPO; for instance, the enzyme 12/15- LOX is found to be highly expressed and converts AA into 12/15-HETE, thus intensifying LPO in the pMCAO mouse model of cerebral I/R. Inhibiting this enzyme with a specific inhibitor, known as LOX Block-1 (LB-1), has proven effective in reducing LPO and significantly decreasing the infarct volume in these models (25). Moreover, studies have indicated that COX-2 and its product, prostaglandin E2 (PGE2), have the potential to exacerbate LPO in cerebral tissues, and that the inhibition of COX-2/PGE2 expression can significantly reduce the impacts of ferroptosis within the brain (35).

The antioxidant system markedly lowers levels of LPO by effectively neutralizing ROS and free radicals, thereby shielding cells from oxidative damage (36). The XC-System/GSH/GPx4 pathway is a critical component in the cellular defense against ferroptosis, a form of regulated cell death characterized by the accumulation of lipid peroxides (37). This protective mechanism centers around the XC-System, which consists of the cystine/glutamate antiporter formed by the proteins SLC7A11 and SLC3A2. These proteins regulate the balance of amino acids inside and outside the cell to synthesize GSH, thereby maintaining antioxidant status (38). In cases of cerebral I/R injury, there is a notable increase in the production of ROS, which can overwhelm the cellular defenses and hinder the functionality of the XC-System/GSH/GPx4 pathway (39). When the components of this pathway are compromised, the body’s overall antioxidant capacity is notably diminished. This decline contributes to an accelerated progression of LPO, which is detrimental to cell viability (40). However, the transcription factor Nrf2 has been identified as a key regulator in this context, as it can stimulate the expression of SLC7A11. When Nrf2 activates SLC7A11, it enhances the activity of the XC-System, leading to an increase in the expression levels of both GSH and GPx4. The subsequent upregulation of these components significantly fortifies the antioxidant defense system, thereby effectively countering lipid peroxidation and inhibiting ferroptosis, illustrating the pathway’s vital role in cellular resilience against oxidative stress (41, 42).

## 3 Inflammation and cerebral I/R

**Figure 2:**
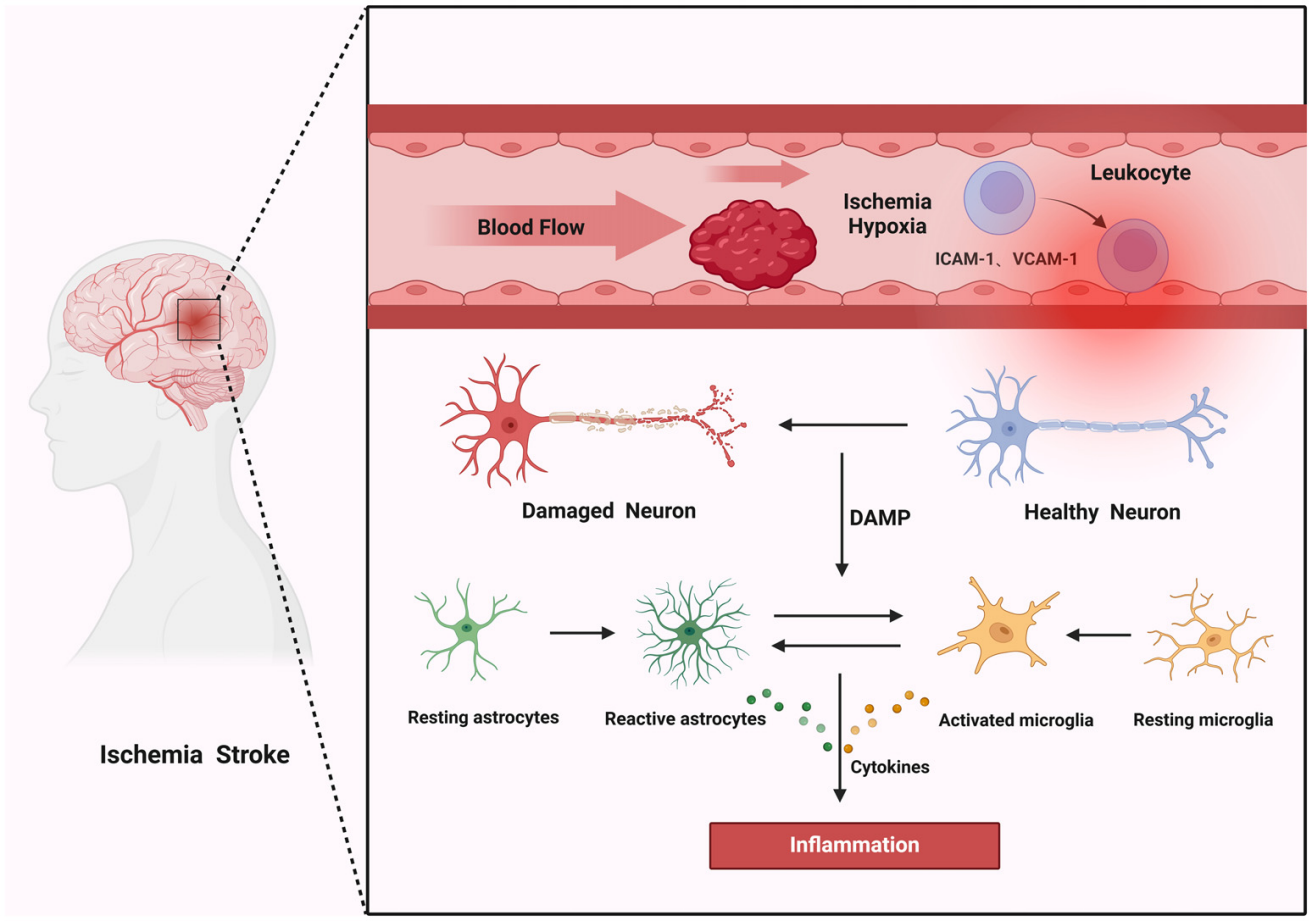
Mechanisms of inflammation in cerebral I/R. Thrombosis induces ischemic hypoxia within blood vessels, activating endothelial cells to express adhesion molecules ICAM-1 and VCAM-1. These molecules attract leukocytes, which rapidly produce pro-inflammatory signals that activate neuroglial cells. Concurrently, ischemic neurons undergo rapid cell death and release damage-associated molecular patterns (DAMPs) that further stimulate neuroglial cells to release inflammatory mediators, resulting in inflammation.

Inflammation begins rapidly during the acute phase of cerebral ischemia, occurring within minutes of the initial event. This inflammatory response can evolve into a significant mechanism of injury within just a few hours and may continue to persist for several days, affecting both intravascular and perivascular territories (43). Shortly after the occlusion of an artery, the inflammatory response is swiftly activated within the vascular lumen. Various factors contribute to this response, including hypoxia, alterations in shear stress, and the generation of ROS. These elements collectively activate the coagulation cascade and stimulate the activation of various cell types, including complement cells, platelets, and endothelial cells. As a result of this cascade, neuronal death occurs, leading to the development of an ischemic core surrounded by a region of reduced blood flow known as the penumbra (44). In the immediate aftermath of ischemia, endothelial cells begin to upregulate the expression of adhesion molecules, such as intercellular adhesion molecule 1 (ICAM-1), vascular cell adhesion molecule 1 (VCAM-1), P-selectin, and E-selectin. The presence of these molecules facilitates the adhesion of leukocytes circulating in the bloodstream to the endothelium. This interaction initiates a cascade of pro-inflammatory signaling that activates immune cells, thereby intensifying neuronal damage (45). The complex interplay of these processes highlights the detrimental impact of inflammation on neuronal survival during the early stages of cerebral ischemia, as depicted in **Figure 2**.

### 3.1 Activation and infiltration of glial cells

Neurons possess a remarkable susceptibility to ischemic conditions, which leads to a swift process of neuronal death. This neuronal demise prompts the release of various endogenous molecules, including ATP, UTP, HMGB1, HSPs, and others that act as DAMPs. These DAMPs are crucial because they are detected by immune cells utilizing pattern recognition receptors such as Toll-like receptors and NOD-like receptors. When these receptors recognize the DAMPs, they trigger the activation of inflammatory signaling pathways. This cascade results in the secretion of additional factors that intensify both localized and systemic inflammatory responses, thereby exacerbating the overall inflammatory condition in the affected tissues (46, 47). Microglia, which serve as the brain’s intrinsic immune defenders, play a critical role in surveilling and sustaining internal equilibrium under normal resting conditions (48). Upon the onset of cerebral ischemia, these microglial cells respond swiftly, transforming their morphology to an amoeboid shape within minutes. They possess the ability to polarize into two distinct phenotypes: the pro-inflammatory M1 and the anti-inflammatory M2 (49, 50). This polarization allows microglia to effectively regulate the inflammatory response, tailoring their actions to the specific needs of the surrounding environment. Following the initial injury to neurons, the damaged cells release a chemokine known as CX3CL1, also referred to as fractalkine. This signaling molecule is recognized by the CX3CR1 receptor expressed on microglial cells, facilitating communication between the damaged neurons and the immune response (51). As a result, the activated microglia migrate to the site of injury, where they actively engulf and eliminate dead cells and cellular debris. This phagocytic activity is vital as it not only helps clear away the remnants of damage but also plays a crucial role in preventing additional cell injury and the further propagation of inflammation in the affected area (52). Astrocytes serve a crucial and multifaceted regulatory function in the context of cerebral I/R. Their roles encompass the modulation of inflammatory responses, the maintenance of the BBB, and the regulation of iron homeostasis (53). Following an episode of cerebral ischemia, the release of cytokines by both neurons and microglia occurs within hours, which triggers the activation and reactive proliferation of astrocytes (54). This rapid response is critical for mitigating brain damage. Activated astrocytes exhibit the ability to polarize into two distinct phenotypes: the pro-inflammatory “A1” and the anti-inflammatory “A2” (55). At the onset of astrocytic activation, the predominant focus is on facilitating a pro-inflammatory response. This initial response aims to swiftly clear areas of brain damage. Simultaneously, it seeks to control excessive inflammation, thereby protecting neurons from sustaining further injury (47).

### 3.2 Inflammatory signaling pathways and factors

**Figure 3:**
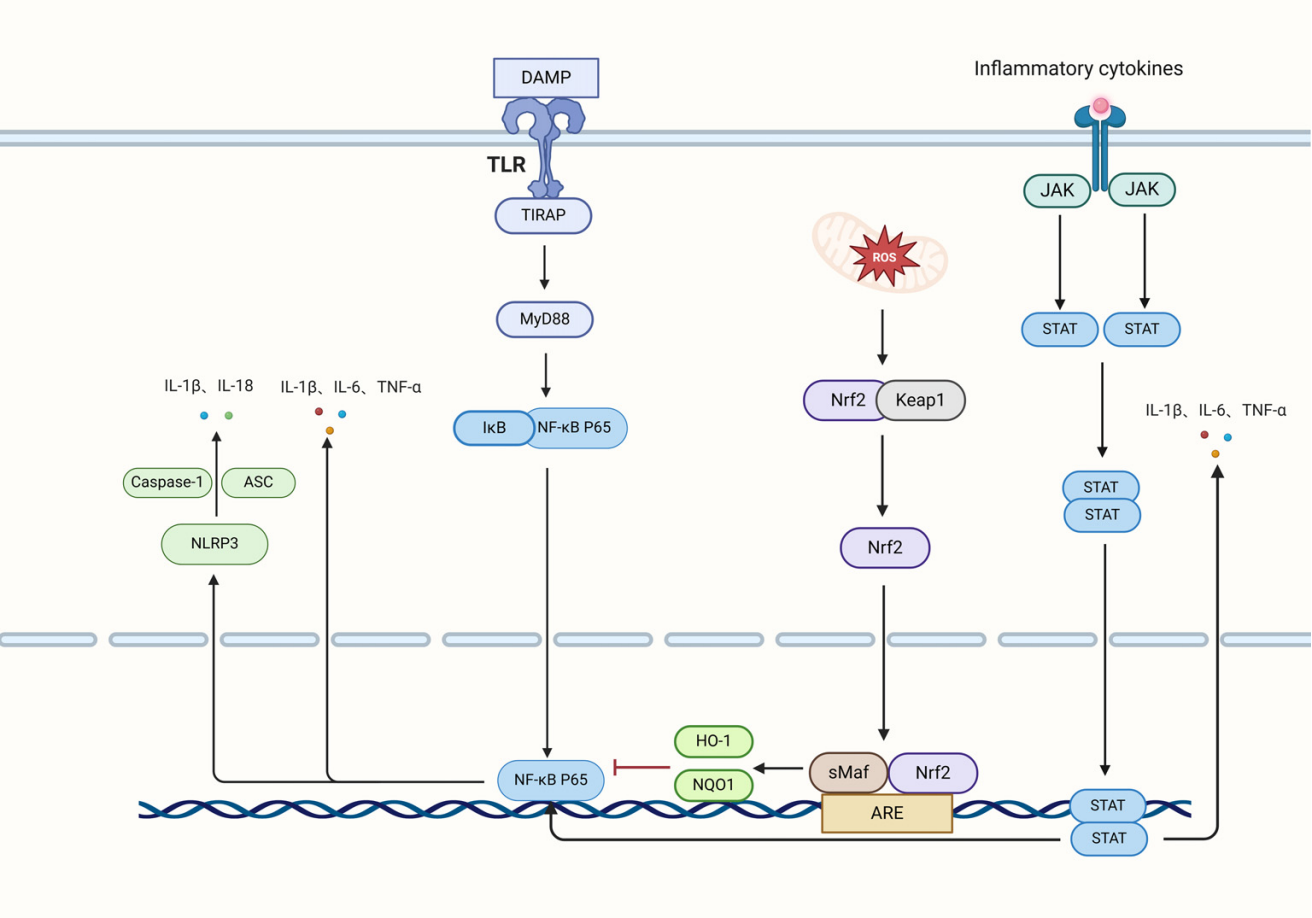
Inflammatory signaling pathways and factors. Neurons release damage-associated molecular patterns (DAMPs) that engage TLRs to transmit signals to TIRAP, subsequently activating MyD88. This activation results in the phosphorylation of IκB, which triggers the translocation of P65 into the nucleus, initiating the transcription and release of the inflammatory cytokines IL-1β, IL-6, and TNF-α. Intracellular oxidative stress activates Nrf2, which dissociates from Keap1, translocates to the nucleus, and induces the transcription of HO-1 and NQO1 via the ARE, thereby inhibiting NF-κB transcription and reducing inflammation. Additionally, NF-κB and oxidative stress activate the NLRP3 inflammasome, which, in conjunction with Caspase-1 and ASC, releases IL-1β and IL-18. The JAK pathway phosphorylates STAT, leading to its dimerization and subsequent entry into the nucleus, where it enhances the transcription of IL-1β, IL-6, and TNF-α, further activating NF-κB transcription.

NF-κB functions as a crucial hub in the process of inflammation, playing a significant role in inflammatory responses, as depicted in **Figure 3**. In the context of cerebral I/R, NF-κB engages with pathways including Nrf2 and NLRP3 to modulate inflammation (56, 57). TLR4, which is mainly found in microglia, triggers the activation of NF-κB via signaling pathways, thereby promoting microglial activation and subsequent inflammatory responses (58, 59). Additionally, the TLR4/NF-κB signaling axis is implicated in mediating inflammatory responses within type A1 astrocytes (60). The anti-inflammatory mechanism involving Nrf2 elevates cellular HO-1 levels through gene transcription driven by antioxidant response elements (ARE) while simultaneously promoting the degradation of IκB-α by the proteasome. This action inhibits the translocation of NF-κB into the nucleus, thereby contributing to the regulation of inflammation (61).

As a key downstream target of NF-κB, the NLRP3 inflammasome functions as an essential mediator in post-ischemic inflammation, leading to a series of inflammatory responses and consequent cell death (62). Gong et al. showed that (63) the activation of the NLRP3 inflammasome occurs initially in microglia following brain I/R injury, with further expression identified in microvascular endothelial cells and, notably, in neurons. Although the primary expression of NLRP3 is found in microglia, there is growing evidence suggesting its activation in astrocytes following I/R injury, where NLRP3 activation might lead to the neurotoxic reprogramming of astrocytes (64). Hence, the modulation of NLRP3 presents considerable therapeutic promise for managing inflammation linked to cerebral I/R.

The JAK2/STAT3 signaling pathway is critical in mediating inflammatory responses, with TNF-α and IL-6 serving as major activators. These cytokines activate microglia and astrocytes in ischemic areas, affecting the expression of various inflammatory factors that contribute to brain damage caused by I/R injury (65, 66). In the setting of cerebral I/R, IL-6 functions as an initiator that fosters STAT3 phosphorylation, setting off a signaling cascade that triggers metabolic reprogramming in reactive astrocytes (67). Additionally, the interplay between the STAT3 and NF-κB pathways can amplify the activation of M1 microglia and reactive astrocytes, thus worsening neuroinflammation (68). — This revision maintains the original ideas while ensuring that phrases are restructured and synonyms are used where appropriate to avoid duplication concerns.

## 4 Crosstalk between ferroptosis and inflammation in cerebral I/R

The relationship between ferroptosis and inflammation in the context of cerebral I/R is intricate and multifaceted. Although these two biological processes can operate independently, they closely interact and exert significant influences on one another. Our focus is on exploring the overlapping mechanisms between ferroptosis and inflammation to develop innovative treatment approaches for brain I/R.

(19). The connection between ferroptosis and neuroinflammation is characterized by its bidirectional nature. Neuroinflammation typically begins shortly after the onset of cerebral ischemia, setting off a cascade of biological responses. In contrast, ferroptosis is primarily observed during the reperfusion phase following ischemic injury. This chronological relationship indicates that neuroinflammation serves as a precursor to ferroptosis in the course of cerebral I/R events (25, 69). As inflammation unfolds, it engenders the activation of glial cells and various related pathways which contribute to the accumulation of iron as well as LPO. These processes compromise the antioxidant defense system’s effectiveness, ultimately precipitating the occurrence of ferroptosis (70). Furthermore, products resulting from LPO that arise during ferroptosis, along with DAMPs released from disrupted cell membranes, play a significant role in activating immune cells. This activation further intensifies the inflammatory response, demonstrating how ferroptosis can exacerbate neuroinflammation (71, 72). The interplay between these two processes establishes a vicious cycle that perpetuates ongoing damage to brain tissue. Within this framework, we delve into the interactions between ferroptosis and inflammation, highlighting emerging mechanisms and potential targets for drug development. Through in-depth research into these mechanisms, we aim to offer novel insights and viable therapeutic strategies for clinical practice, thus enhancing the prognosis for patients suffering from brain I/R.

### 4.1 Inflammation-induced ferroptosis

**Figure 4:**
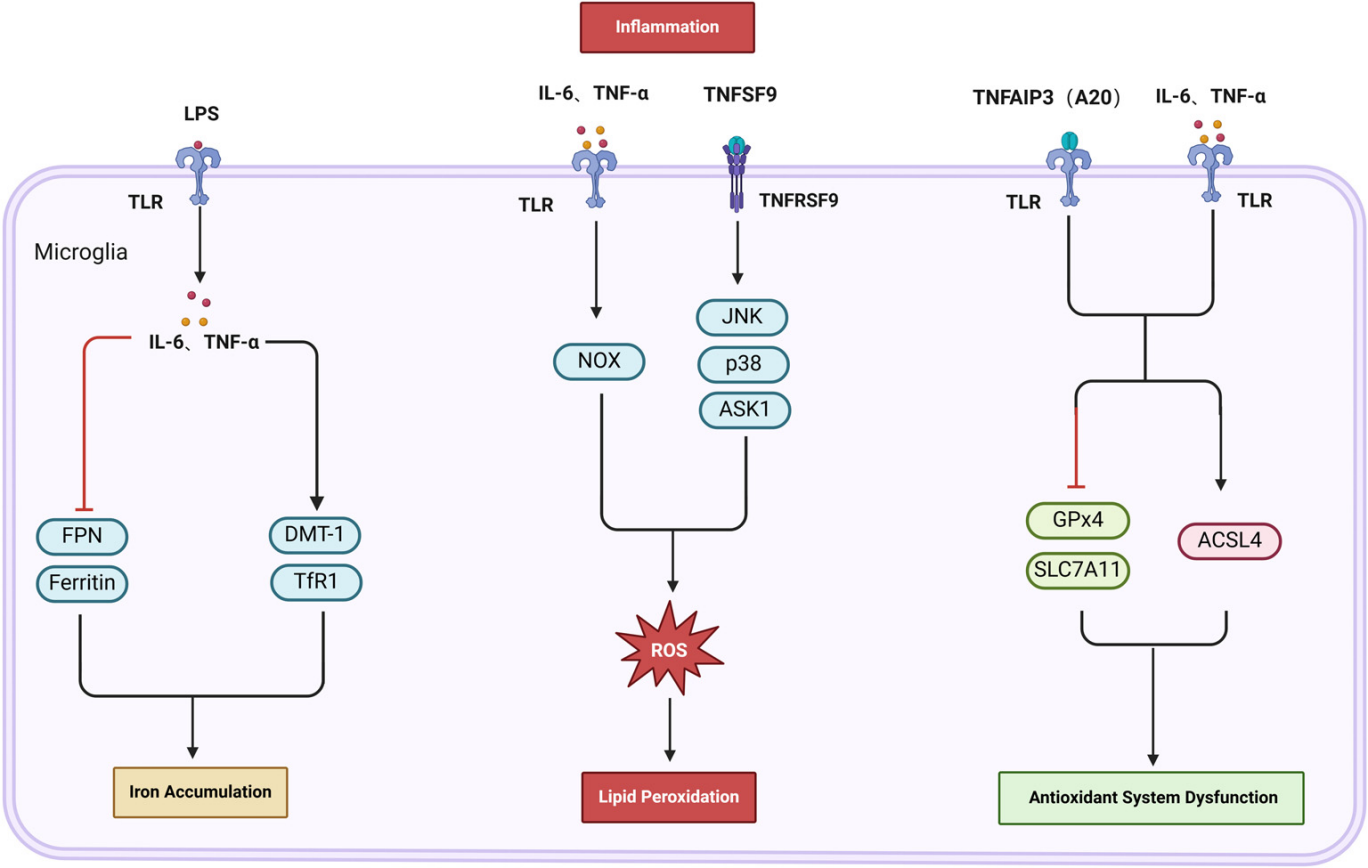
The activation of glial cells and associated signaling pathways leads to iron accumulation, LPO, and decreased activity of the antioxidant system, forming critical pathways for inflammation-induced ferroptosis. Lipopolysaccharide (LPS) activates microglia, which secrete IL-6 and TNF-α, leading to the upregulation of DMT-1 and TfR1, while downregulating ferritin and FPN. This cascade results in increased iron levels. Moreover, Tumor necrosis factor superfamily member 9 (TNFSF9) and NADPH oxidase (NOX) can induce lipid peroxidation (LPO) by generating reactive oxygen species (ROS). Furthermore, TNF-induced protein 3 (TNFAIP3, or A20) in conjunction with IL-1β and IL-6, influences the antioxidant system’s activity. This is achieved by upregulating ACSL4 and downregulating the expression of GPX4 and SLC7A11, thereby intensifying LPO.

In cerebral I/R, the activation of glial cells and associated signaling pathways leads to iron accumulation, LPO, and decreased activity of the antioxidant system, forming critical pathways for inflammation-induced ferroptosis (73), as depicted in **Figure 4**.

#### 4.1.1 Role of glial cells in ferroptosis

Glial cells serve as facilitators for the accumulation of iron triggered by inflammation in cerebral I/R (74). They are vital for iron metabolism in the brain; while microglia store considerable quantities of iron, astrocytes have the ability to transfer this iron to adjacent neurons, effectively intertwining iron metabolism with inflammatory processes (75, 76). The presence of lipopolysaccharide (LPS) prompts microglia to generate inflammatory cytokines, including IL-6 and TNF-α, which not only activate these cells but also enhance the expression of DMT1 and downregulate FPN, thereby increasing iron uptake and retention within microglia (77). Thus, inhibiting IL-6 and TNF-α induced upregulation of DMT1 and downregulation of FPN in microglia can mitigate iron accumulation. Research indicates that LPS activates M1 microglia, increasing TfR and reducing FTH, thereby elevating iron levels. Conversely, stimulation with IL-4 activates M2 microglia, which leads to an increase in both TfR and FTH, sequestering free intracellular iron and reducing iron-associated toxicity (78). Accordingly, decreasing M1 and enhancing M2 microglia expression can lower iron accumulation via adjustments in TfR and FTH. Additionally, the LPS-mediated IL-6 expression in microglia triggers STAT3 phosphorylation, signaling astrocytes to elevate hepcidin levels, which leads to a decrease in FPN1 expression in astrocytes and, subsequently, increased iron accumulation (79). Research on post-ischemic conditions by Davaanyam et al. demonstrates that (80) HMGB1 in astrocytes exacerbates inflammation and enhance hepcidin levels through TLR4 and CXCR4 signaling, resulting in an increase in iron levels and subsequent ferroptosis. Thus, by inhibiting the expression of the IL-6/STAT and HMGB1/TLR4 pathways in glial cells, FPN levels can be increased, reducing iron accumulation and consequently mitigating ferroptosis. Experimental findings indicate that (81) exosomes derived from M1-polarized BV2 microglia exhibit higher expressions of IL-1β, IL-6, and TNF-α. These cytokines promote iron release by downregulating FTH1 expression, leading to iron accumulation in neuronal cells and exacerbating neuronal injury. In contrast, exosomes from M2 microglia transport miR-124-3p to HT22 cells exposed to OGD/R, targeting NCOA4 and thereby inhibiting ferroptosis linked to iron autophagy, ultimately reducing iron accumulation in HT22 cells (82). Although the exact components of the exosomes remain unidentified, these findings indicate that exosomes can mitigate iron accumulation by inhibiting M1 microglial cells and enhancing the anti-inflammatory M2 microglial phenotype, providing a new approach to treat inflammation-driven iron accumulation.

Furthermore, the stimulation of glial cells by inflammation leads to the production of ROS and worsens LPO, ultimately resulting in ferroptosis (74). M1 microglial cells, by activating NADPH oxidase (NOX) through cytokines like TNF-α and IL-1β, produce substantial amounts of ROS, which intensify LPO. Consequently, targeting NOX to inhibit ROS generation could serve as a potential strategy to prevent ferroptosis induced by microglia-enhanced LPO (83, 84). Subsequently, Li et al. (85) found that tumor necrosis factor ligand superfamily member 9 (TNFSF9) engages with TRAF1 to activate mouse BV2 microglia, promoting ROS generation via the JNK, ASK1, and p38 signaling pathways. This activation reduces SLC7A11 and GPx4 activities, thus promoting ferroptosis; however, this process can be reversed by inhibiting the TNFSF9/TRAF1 signaling cascade. Therefore, investigating TNFSF9 to curb inflammation-driven microglial ferroptosis shows considerable potential. Reducing the activity of the antioxidant system represents another pathway by which inflammation exacerbates ferroptosis. Liao et al. revealed that (86) the M1 microglial phenotype encourages the discharge of IL-1β and IL-6, while simultaneously inhibiting IL-10. This imbalance causes reduced levels of GSH and GPx4, which aggravates iron-mediated cellular death and neurological harm after OGD/R. The TNF-induced protein 3 (TNFAIP3, or A20), a significant regulator of inflammatory responses, negatively regulates microglial polarization via the TLR/NF-κB pathway (87). Liu et al. highlighted that (88) in microglia with A20 knockdown induced by RSL3, the heightened levels of IκBα/NFκB/iNOS resulted in increased inflammation, whereas upregulation of GPx4 and SLC7A11 along with downregulation of ACSL4 decreased the microglial susceptibility to ferroptosis. This observation may appear contradictory to the belief that inflammation intensifies ferroptosis; however, it suggests that A20 plays a dual role in governing both inflammatory responses and ferroptosis in microglia. Microglia that without A20 knockdown showed more pronounced ferroptosis, indicating that knocking down A20 expression could help mitigate ferroptosis triggered by microglial activation. This indicates the need to delve further into the specific molecular mechanisms at play between A20 knockdown and the inflammatory and ferroptosis responses in microglial cells, evaluating A20’s therapeutic target potential. Additionally, IL-6 can activate astrocytes through the STAT3 pathway, leading to a downregulation of GPx4 and FSP1 and promoting ferroptosis, a process significantly countered by the anti-inflammatory agent IL-10 (89).

Neuroglial cells play a crucial role in inflammation-induced ferroptosis. By modulating the activation state of these cells and targeting iron accumulation mechanisms mediated by TNF-α, IL-6/STAT, and HMGB1/TLR4, strategies to inhibit ROS production by M1-type microglial cells and restore the activities of GPX4, SLC7A11, and ACSL4 can effectively prevent ferroptosis. Furthermore, these pathways may represent new approaches to treating inflammation-induced ferroptosis. For example, suppressing TNFSF9 expression can downregulate the ROS produced by inflammation, thereby mitigating ferroptosis. Additionally, knocking down A20 reduces both inflammation and ferroptosis in microglia. Moreover, inflammation-related pathways and factors are also significant in governing the interplay between inflammation and ferroptosis.

#### 4.1.2 Impact of inflammatory pathways and factors on ferroptosis

Inflammatory pathways are essential in facilitating ferroptosis during cerebral I/R events (70). NF-κB serves as a key regulator of inflammation and triggers ferroptosis upon its activation. For example, within the HT22 cell model, activation of the STAT3/NF-κB pathway leads to elevated Fe2^+^ levels and ACSL4 expression, while concurrently inhibiting SLC7A11 and GPX4 activities, ultimately inducing ferroptosis (90). Conversely, the Nrf2 signaling pathway plays a protective role by blocking the transcription of NF-κB p65, which helps reduce inflammation and decrease the incidence of ferroptosis (91). These results highlight the protective function of Nrf2 against ferroptosis; however, there is also evidence indicating that (92, 93) the Nrf2/HO-1 pathway might inadvertently contribute to iron accumulation as it metabolizes heme into CO, biliverdin, and iron (94). Research by Wang et al. has demonstrated that (95) inhibiting HO-1 with minocycline during cerebral I/R can mitigate iron accumulation in microglia and subsequent ferroptosis. While the Nrf2/HO-1 pathway is generally viewed as advantageous for preventing ferroptosis, it may also have negative repercussions; therefore, additional research is necessary to develop strategies aimed at preventing iron buildup mediated by this pathway.

As a pathway downstream of NF-κB, the NLRP3 inflammasome plays a role in inducing ferroptosis. The activation and assembly of NLRP3 lead to the release of IL-1β and IL-18, which subsequently affect the activities of the Nrf2/HO-1 pathway and the xc-GSH-GPX4 system, contributing to iron accumulation (96). Research conducted by Wang et al. revealed that (97) the knockout of NLRP3 in mice significantly diminished brain infarct volume, along with a reduction in the expression levels of IL-18 and IL-1β when compared to wild-type MCAO mice. Furthermore, the absence of NLRP3 resulted in increased levels of GSH and GPX4, while decreasing Fe2^+^ and MDA concentrations, likely associated with the Nrf2/HO-1 pathway.

HMGB1, recognized as a damage-associated molecular pattern, is released in response to cellular damage and can intensify inflammatory reactions (98). In the setting of cerebral I/R, the increased levels of HMGB1 inhibit the expression of both Nrf2 and GPx4, thus affecting the efficacy of the antioxidant system and promoting ferroptosis. Therefore, blocking HMGB1 expression could potentially elevate the activities of both Nrf2 and GPx4, lessening the occurrence of ferroptosis (99, 100).

These results highlight that during cerebral I/R, inflammation mediated by NF-κB, NLRP3, and HMGB1 can trigger ferroptosis. Although the upregulation of HO-1 might result in iron accumulation, interventions targeting Nrf2 generally suppress inflammation and diminish ferroptosis. Thus, promoting Nrf2 while inhibiting NF-κB, NLRP3, and HMGB1 could be beneficial in addressing inflammation-induced ferroptosis. The relationship between inflammation and ferroptosis is reciprocal, as ferroptosis can also provoke inflammatory responses, thereby worsening ischemic damage in cerebral I/R.

### 4.2 Ferroptosis exacerbates inflammatory responses

**Figure 5:**
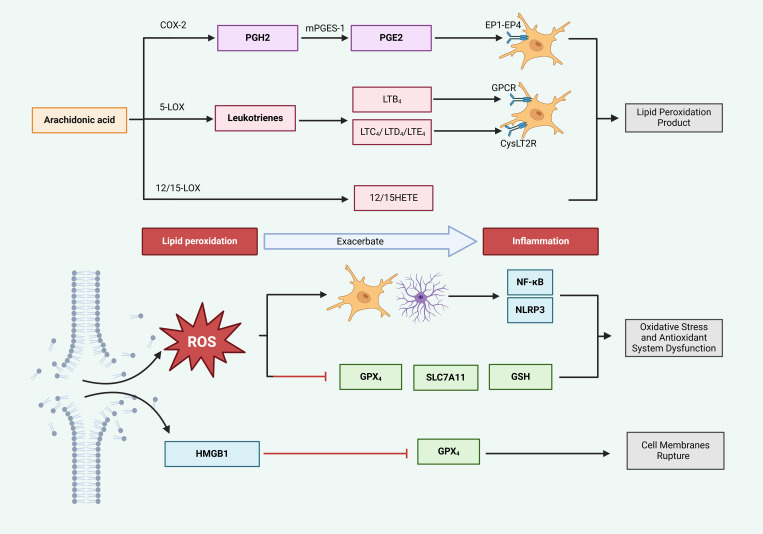
Ferroptosis exacerbates inflammatory responses. Cyclooxygenase (COX) and lipoxygenase (LOX) enzymes catalyze arachidonic acid (AA) to produce lipid peroxides, which trigger inflammation. Specifically, COX-2 converts AA into prostaglandin H2 (PGH2), which is subsequently transformed into prostaglandin E2 (PGE2) by microsomal prostaglandin E synthase-1 (mPGES-1). PGE2 interacts with EP1-EP4 receptors, stimulating glial cells and provoking inflammatory responses. Concurrently, 5-LOX metabolizes AA into leukotrienes; leukotriene B4 (LTB4) interacts with G protein-coupled receptors (GPCR), while LTC4, LTD4, and LTE4 engage with CysLT2 receptors, collectively stimulating glial cells and promoting inflammation. In the lipid peroxidation (LPO) process, ROS are generated, activating glial cells and inducing inflammation via the NF-κB and NLRP3 pathways. Additionally, ROS suppress the activity of GPx4, SLC7A11, and GSH, thereby accelerating the LPO-mediated generation of ROS and indirectly intensifying inflammation. Furthermore, HMGB1, released from ruptured cell membranes, further suppresses GPx4 activity, elevates ROS levels, and exacerbates inflammation.

The buildup of iron ions enhances oxidative stress, leading to a significant increase in the generation of lipid peroxides and ROS. These oxidative byproducts not only inflict direct harm to cellular components but also trigger and escalate inflammatory responses via multiple pathways, which in turn inflicts additional damage on brain tissue (71), as demonstrated in **Figure 5**.

#### 4.2.1 LPO products activate inflammation

Products of LPO, catalyzed by both COX and LOX, display properties that promote inflammation (35, 101). The COX-2/PGE2 pathway is acknowledged as a key factor in ferroptosis triggered by cerebral I/R (102). In this process, COX-2 facilitates the transformation of AA, which results in heightened levels of PGE2. The resulting PGE2 binds to various prostaglandin receptors (EP1-EP4), leading to the increased migration of microglia and neutrophils, thus intensifying the inflammatory response. Furthermore, the generation of ROS may activate COX-2, resulting in a self-reinforcing cycle (103). Therefore, COX-2/PGE2 signifies a promising target for addressing inflammation caused by ferroptosis-related products.

Moreover, 5-LOX, a specific variant of LOX, has the capability to trigger ferroptosis and generate harmful LPO byproducts, positioning it as a crucial target for inhibiting this pathway (101). This enzyme facilitates the peroxidation of AA, resulting in the creation of inflammatory substances, including leukotrienes (LTB4, LTC4, LTD4, and LTE4). LTB4 subsequently engages their G-protein-coupled receptors. The engagement of these receptors initiates the migration and activation of immune cells, thus enhancing local inflammation (104). Additionally, LTC4, LTD4, and LTE4 provoke inflammatory reactions through the activation of cysteinyl leukotriene receptors (CysLTR), which may activate the NF-κB signaling cascade, further promoting neuronal damage and inflammation, as well as encouraging M1 polarization within microglia (105, 106). By targeting the lipid peroxidation products linked to 5-LOX, leukotrienes, and their corresponding receptors, it may be possible to mitigate the infiltration of immune cells (such as microglia and neutrophils) and downregulate the expression of NF-κB, thereby lessening inflammatory responses.

The heightened activity of 12/15-lipoxygenase (12/15-LOX) is significantly linked to the process of ferroptosis, with its lipid peroxidation derivatives, specifically 12-HETE and 15-HETE, being associated with ischemic stroke (107). Research into the inflammatory mechanisms related to ischemic stroke has shown that (108) 12/15-LOX can trigger the activation of NLRP1 and NLRP3, which subsequently leads to the secretion of IL-1β. This release promotes inflammation and contributes to brain injury in pMCAO mice. Through these LPO processes, PUFAs are ultimately converted into the peroxidation product 4-hydroxynonenal (4-HNE) (109). Due to its pro-inflammatory nature, 4-HNE activates microglia through the TLR4/NF-κB signaling pathway, leading to an increased expression of the inflammatory factors IL-1β and TNF-α, thus intensifying neuroinflammation. Therefore, the LPO product 4-HNE represents a potential therapeutic target for inflammation caused by ferroptosis (110). Furthermore, ACSL4 plays a role in both ferroptosis and inflammation by converting free arachidonic acid (AA) into arachidonyl-CoA, thereby enhancing ferroptosis (111). Simultaneously, ACSL4 is capable of activating microglial NF-κB pathways, resulting in the production of cytokines such as TNF-α, IL-6, and IL-1β. Importantly, the silencing of ACSL4 reverses the expression levels of these inflammatory markers (34, 112). Although these processes seem to be interconnected, treatment with RSL3 did not lead to an increase in inflammatory cytokines in LPS-stimulated microglia that were silenced for ACSL4 (112). This observation implies that while the inhibition of ACSL4 may alleviate ferroptosis and associated neuroinflammation, the link between reduced neuroinflammation and ferroptosis may be limited, suggesting that further investigations are necessary to determine whether ACSL4 can be effectively targeted to treat inflammation induced by ferroptosis and to clarify the relationship between these processes.

LPO can produce substantial quantities of ROS, which activate reactive astrocytes and microglia. This activation triggers the transcription of inflammatory cytokines such as IL-6, TNF-α, IL-1β, and IL-18 through various pathways like NF-κB and NLRP3, thereby initiating an inflammatory response (113). The NOXfamily is considered a key contributor to cellular ROS generation (114). Research conducted by Yauger et al. revealed that (115) iron exposure results in ROS production within microglia; however, the use of NOX2 and NOX4 inhibitors diminishes this iron-induced ROS production, highlighting NOX as a potential target for the suppression of microglial activation triggered by ferroptosis.

Excessive ROS can surpass the capabilities of the antioxidant system, resulting in swift dysregulation and promoting the unrestrained advancement of LPO (116). As a result, bolstering the antioxidant system can significantly alleviate ferroptosis and inflammation induced by LPO. However, during the process of ferroptosis, the antioxidant system tends to become rapidly exhausted, indicating the need for its reinforcement to control ROS produced and to mitigate inflammation, particularly through the xc−/GSH/GPX4 pathway (117). The Nrf2 signaling pathway has the ability to enhance the expression of GPX4, SLC7A11, and GSH, subsequently improving the functionality of the antioxidant system and lowering inflammation brought about by ROS (118). In addition, the RNA-binding protein Pumilio 2 (PUM2) binds to PUM-binding elements (PBEs) found in the 3’ untranslated regions (UTRs) of particular mRNAs, and it has been demonstrated to worsen neuroinflammation caused by cerebral I/R by facilitating ferroptosis (119). In their study, Liu et al. established that (120) PUM2 reduces the expression of SIRT1, which in turn restrains the activity of the downstream target SLC7A11, leading to increased levels of ACSL4 and TFR1, while decreasing FTH1 and GPX4 levels. This sequence of events contributes to ferroptosis and worsens neuroinflammation as well as brain injury linked to cerebral I/R. Thus, targeting PUM2 expression may produce positive outcomes, as the suppression of the SIRT1/SLC7A11 pathway by PUM2 is associated with ferroptosis and increased neuroinflammation.

#### 4.2.2 Activation of inflammatory signaling following cellular injury

Excessive LPO leads to the disruption of neuronal cell membranes, which facilitates the release of DAMPs like HMGB1 into the extracellular environment. These DAMPs subsequently stimulate immune cells through the TLR/NF-κB signaling pathway, resulting in neuroinflammation (121). Research by Wen et al. established that (122) HMGB1 released during the process of ferroptosis encourages the production of inflammatory cytokines, suggesting that targeting the regulation of HMGB1 could be an effective strategy for managing inflammation linked to ferroptosis.

The pathways through which ferroptosis triggers neuroinflammation involve lipid peroxidation-derived products such as PGE2 and leukotrienes, along with ROS and HMGB1 released as a result of cell membrane damage. These mediators can activate neuroglial cells, worsening inflammation via pathways like TLR/NF-κB, NLRP3, and COX-2/PGE2. Consequently, targeting pathways such as COX-2/PGE2, 5-LOX/leukotrienes, 12/15-LOX, NOX/ROS, and HMGB1 may alleviate neuroinflammation associated with ferroptosis. Furthermore, inhibiting the PUM2-induced downregulation of SIRT1 could present a novel mechanism for addressing inflammation resulting from ferroptosis, although the specific mechanisms by which PUM2 facilitates inflammation via ferroptosis necessitate additional exploration. Additionally, targeting the ferroptosis byproduct 4-HNE represents another therapeutic strategy for inflammation; however, no drugs have yet been confirmed effective in this context. Therefore, ongoing research is essential to elucidate specific pathways for targeting 4-HNE in treating brain ischemia/reperfusion (I/R) associated with ferroptosis and inflammation.

## 5 Therapeutic mechanisms

As illustrated in **Table 1**.

**Table 1:** Therapeutic mechanisms for ferroptosis and inflammatory crosstalk in cerebral ischemia-reperfusion.

Therapeutic Mechanisms	Name	Pathway	Effects on cytokine levels	Reference
Inhibiting inflammation-induced ferroptosis	Glycyrrhizin	HMGB1/GPX4↓	TNF-α, IL-6, IL-1β↓	(123, 124)
	Icariin II	Nrf2/OXPHOS/NF-κB↓	TNF-α, IL-6, IL-1β, ROS, MDA↓ SIRT5, NADPH/NADP, SOD2, GPX_4_↑	(91, 125)
	Dimethyl fumarate (DMF)	NRF2/NF-κB↓	IL-1β, TNF-α, IL-6↓ GSH, SOD, GPX_4_↑	(126)
	sulforaphane (SFN)	NRF2/NF-κB↓	Occludin, ZO1↑ IL-18, IL-1β, Fe2^+^↓, GSH, GPX4↑	(127)
	Oxysophoridine (OSR)	TLR/p38 MAPK↓	Fe^2+^, ACSL4, TFR1↓ FTH1, GPX4↑	(128–131)
	Salvia miltiorrhiza (Active ingredients are Salvianolic Acid B and Tanshinone IIA)	IL-6/STAT3↓	TNF-α, ROS, ACSL4↓ FPN1, GPX4↑	(132–135)
	Nao Tai Fang (Active ingredients are Ferulic Acid, Ligustilide, Astragaloside IV, and Tetramethylpyrazine)	M1 microglia, IL-6/STAT3↓ M2 microglia, BMP6/SMADs↑	IL-1β, IL-6, Fe^2+^, ROS↓ IL-10, GSH, GPX_4_↑	(86, 136, 137)
Inhibiting ferroptosis-induced inflammation	Caffeic Acid (CA)	COX-2/PGE2, 5-LOX NF-κB p65↓ Nrf2↑	ROS, TNF-α, IL-6, IL-1β iNOS↓	(138–141)
	ADA-409-052	COX-2/PGE2, Microglial activation, Macrophage↓	TNF-α, IL-6, IL-1β↓ GSH, GPx4↑	(142, 143)
	Edaravone	5-LOX↓ Nrf2/FPN↑	Fe^2+^, MDA, IL-6, IL-1β, TNF-α, leukotriene↓ GSH↑	(144–147)
	Panax notoginseng saponins (PNS)	Nrf2↑	Fe2^+^, ACSL4, SLC7A11, IL-6, IL-1β, TNF-α↓ GSH, GPX_4_ ↑	(148, 149)
	Gastrodia elata polysaccharides	NRF2/HO-1↑ HMGB1, NLRP3↓	Fe^2+^, ROS, IL-1β, IL-6 TNF-α↓	(150, 151)
	Srs11-92 (AA9)	NRF2/NF-κB↓ NRF2/HMGB1↓	GSH, GPX_4_↑	(152)
Other Therapeutic Strategy	Enriched environments (EE)	IL-6/STAT3, Astrocytic activation↓	ROS, Fe2^+^ ↓, GPX4↑	(153–156)
Exosomes	ADSC-Exo	Fxr2/Atf3/Slc7a11, M2 microglia↑ M1 microglia↓	Fe^2+^, hepcidin, TfR1, DMT1, TNF-α, IL-6, IL-1β, ACSL4↓ FPN1, GPX4, HIF-1α↑ IL-1β, IL-2, IL-6, IL-12 p70, IL-23, TNF-α, MDA ROS, Fe^2+^↓ IL-10, TGFβ1, IL-4, IL-13↑	(159)
	SRC-3-exo	Ferroptosis, Microglial activation, Astrocytic activation↓	MDA, IL-1β, IL-6, TNF-α↓ GSH/GSSG↑	(160)
	MSCs-EVs	MSCs-EVs/Fer-1↑ GPX4/COX-2/PGE2↓	TNF-α, IL-1β↓	(161)

↑ is upregulating ↓ is downregulating.

### 5.1 Modulating inflammation-induced ferroptosis

Glycyrrhizin, a key ingredient found in the traditional herb licorice, acts as a blocker of HMGB1 and is utilized in treating cerebral I/R (123). Research by Zhu et al. demonstrated that (124) glycyrrhizin lowers the levels of TNF-α, IL-6, and IL-1β through the HMGB1/GPX4 pathway, effectively preventing neuroinflammation-related neuronal ferroptosis and alleviating damage from cerebral ischemic hypoxia. Icariin II, which is the main active component of the traditional herb horny goat weed, possesses anti-inflammatory, antioxidant, anti-ferroptotic, and anti-apoptotic attributes in the context of cerebral I/R (125). According to a study by Gao et al. (91), prior treatment with icariin II in astrocytes notably enhances Nrf2 expression, which subsequently reduces the secretion of IL-1β, IL-6, and TNF-α via the OXPHOS/NF-κB pathway. This mechanism leads to diminished production of mitochondrial ROS, MDA, and iron following ischemic stroke, while also increasing the ratios of NADPH/NADP, enhancing SOD2 activity, elevating GPX4 levels, and boosting SIRT5 activity, thereby averting LPO-triggered ferroptosis. These results suggest that icariin lessens inflammation-related ferroptosis through the Nrf2/OXPHOS/NF-κB pathway, offering significant neuroprotection after ischemic stroke. Findings from Zhang et al. indicate that (126) dimethyl fumarate (DMF), an activator of Nrf2, promotes the upregulation of IκBα while inhibiting the NF-κB pathway, resulting in increased expression of crucial ferroptosis mediators, including HO-1, NQO1, and GPx4, thereby safeguarding cells from oxidative stress and ferroptosis. Additionally, recent studies on cerebral I/R have demonstrated that (127) the Nrf2 activator sulforaphane (SFN) enhances the levels of Occludin and ZO1, which helps maintain the integrity of the blood-brain barrier (BBB). SFN also leads to decreased levels of NF-κB, IL-18, and IL-1β, diminishes inflammation, alleviates Fe2^+^ concentrations, and boosts GSH and GPX4 levels, ultimately mitigating ferroptosis and reducing iron buildup through the enhancement of the antioxidant defense system. Oxysophoridine (OSR), a bioactive alkaloid sourced from sophora flowers, displays antioxidant and anti-inflammatory characteristics while providing protection against cerebral I/R damage in mice (128, 129). The TLR/p38 MAPK pathway is acknowledged for its crucial role in modulating inflammation during cerebral I/R (130). Recent research indicates that (131) OSR restrains the activity of the TLR4/p38 MAPK signaling pathway in rats experiencing MCAO, resulting in a decrease in Fe2^+^ accumulation and a marked downregulation of ACSL4 and TFR1 protein levels. Simultaneously, OSR upregulates levels of FTH1 and GPx4, thereby countering ferroptosis. Salvia miltiorrhiza, commonly known as Danshen, is extensively employed in the treatment of cerebrovascular disorders owing to its properties that include anticoagulant, antioxidant, anti-inflammatory, and anti-apoptotic effects (132). It comprises active compounds like salvianolic acid B and tanshinone IIA, although their precise constituents remain to be thoroughly elucidated (133, 134). In a mouse model of tMCAO, the administration of Danshen markedly suppresses the activation of microglia and astrocytes, leads to a downregulation of TNF-α and IL-6 expressions, as well as the phosphorylation of STAT3. This sequence of events mitigates neuroinflammation-induced upsurge in ROS and ACSL4 levels, while also decreasing GPX4 and FPN1, thus providing an inhibition of ferroptosis, sustaining synaptic integrity, and lessening neuronal death. These actions illustrate lasting neuroprotection in the aftermath of ischemic brain damage (135). Nao Tai Fang (NTF), which is made up of traditional Chinese medicinal components like Astragalus, Chuanxiong, Dilong, and Jiangcan, has been found to safeguard rats against neuronal ferroptosis triggered by acute cerebral ischemia (136, 137). Research by Liao et al. revealed that (86) key active ingredients in Nao Tai Fang, such as ferulic acid, Ligustilide, astragaloside IV, and tetramethylpyrazine, impede M1 microglia polarization while encouraging M2 polarization. This adjustment results in a reduction of IL-1β and IL-6/STAT3 expression alongside an increase in IL-10 secretion. Additionally, NTF attenuates Fe2^+^ buildup and the excessive production of ROS induced by LPO through the BMP6/SMADs signaling pathway associated with iron metabolism, all while boosting the levels of GSH and GPx4, thereby inhibiting ferroptosis.

### 5.2 Modulating inflammation caused by ferroptosis

Caffeic Acid (CA), a naturally occurring phenolic compound generally found in medicinal plants, serves as an inhibitor of COX-2/PGE2 (138). In studies involving the pMCAO rat model, CA has been shown to hinder the activation of COX-2 induced by ferroptosis and reduce the associated increase in ROS, thus preventing the activation of both microglia and astrocytes. Moreover, CA diminishes the release of pro-inflammatory cytokines such as TNF-α, IL-6, and IL-1β, effectively reversing the damage resulting from cerebral I/R (139). Furthermore, it has been shown that CA acts as a 5-LOX inhibitor, mitigating inflammation related to brain I/R (140). In rat models, CA administration leads to a reduction in 5-LOX expression, downregulating NF-κB p65 activation, and decreasing the release of iNOS (141). Researchers, including Keuters (142), have discovered a novel small molecule, ADA-409-052, which features an arylthiazine framework and inhibits LPO, thereby preventing neuronal cell death caused by GSH depletion or GPx4 inhibition. In addition, ADA-409-052 curtails the pro-inflammatory activation of BV2 microglia, lessens the release of TNF-α, IL-6, and IL-1β, and provides protection to N2a neuronal cells from inflammation-induced cell death mediated by RAW 264.7 macrophages. Similar to well-established ferroptosis inhibitors like Fer-1, which can alleviate neuroinflammation (143), ADA-409-052 interacts with the COX-2/PGE2 pathway to target ferroptosis, helping to reduce both severe neuronal injury and inflammation, making it a potential therapeutic approach for neurological disorders (142). Edaravone represents a highly effective neuroprotective agent that functions as a potent scavenger of free radicals, thereby inhibiting both ferroptosis and inflammation associated with ischemic stroke (144, 145). Recent studies have shown that following cerebral I/R, there is a notable decrease in the expression levels of Nrf2 and FPN; however, these alterations can be reversed with edaravone treatment (146). This therapeutic intervention not only diminishes the concentrations of Fe2+ and MDA within the brain tissue of MCAO/R rats but also elevates GSH levels, suppresses ferroptosis, and reduces the concentrations of inflammatory markers such as IL-6, IL-1β, and TNF-α. These observations suggest that edaravone mitigates cerebral I/R injury through the activation of the Nrf2/FPN signaling pathway, which leads to the inhibition of both ferroptosis and inflammation. Nonetheless, additional research is necessary to clearly define the relationship between reduced inflammation and the Nrf2/FPN pathway. Moreover, edaravone lowers extracellular levels of leukotrienes by blocking the 5-LOX pathway, thus safeguarding SD rats and PC12 cells from inflammatory damage induced by ischemia (147). Panax notoginseng saponins (PNS), which are extracted from the Chinese medicinal herb Panax notoginseng, display various beneficial effects including antioxidation, anti-inflammation, anti-apoptosis, and the promotion of angiogenesis (148). In MCAO/R rat models, the administration of PNS activates the Nrf2 signaling pathway, suppresses Fe2^+^ and ACSL4 expression, decreases levels of LPO, and enhances the concentrations of GSH, GPX4, and SLC7A11, thereby strengthening the antioxidant defense system, inhibiting ferroptosis, and subsequently reducing the levels of IL-6, IL-1β, and TNF-α to mitigate neuroinflammation and address cerebral I/R (149). Moreover, polysaccharides from Gastrodia elata, a traditional Chinese herb, exhibit properties such as antioxidant, anti-inflammatory, and anti-ferroptotic effects (150). In models of MCAO mice, treatment with these polysaccharides leads to a marked activation of the NRF2/HO-1 signaling pathway, a decrease in the harmful buildup of Fe2^+^, and an enhancement of GSH and GPx4 activities. By obstructing LPO, the treatment helps avert damage to cell membranes and the ensuing release of HMGB1 and ROS, thus mitigating neuronal ferroptosis. This mechanism is associated with the stimulation of the NLRP3 pathway, which subsequently triggers the release of pro-inflammatory cytokines, including IL-1β, IL-6, and TNF-α, ultimately reducing neuroinflammation (151). These findings highlight the protective effects of Gastrodia elata polysaccharides against ferroptosis and inflammation induced by cerebral ischemia/reperfusion. The Ferrostatin-1 derivative, Srs11-92 (AA9), has been shown to impede iron accumulation and ROS generation, boost GPX4 and Nrf2 expression, and inhibit the levels of HMGB1 and NF-κB p65 within the hippocampal region. This intervention led to a reduction in infarct size, lessened neuronal injury, and improved neurological outcomes in the MCAO/R mouse model. Conversely, the Nrf2 inhibitor ML385 negated the protective effects of AA9, suggesting that AA9 alleviates neuronal ferroptosis and neuroinflammation induced by OGD/R through the Nrf2 pathway (152).

### 5.3 Other therapeutic strategies

Enriched environments (EE) serve as a rehabilitation approach for stroke patients, aimed at offering greater space, innovative game tools, and more social interactions. This method fosters improved sensory, cognitive, motor, and social engagement, which supports neuronal reorganization and functional recovery after injury, while also enhancing the central immune response (153, 154). Research indicates that EE can reduce the activation of IL-6-induced STAT3 phosphorylation in MCAO rats, leading to lower hepcidin levels in astrocytes, reduced TfR1 and DMT1 expression, increased FPN1 activity, and a decrease in iron accumulation due to neuroinflammation (155). Moreover, EE effectively reduces iron accumulation and mitigates the downregulation of GPX4 induced by inflammatory cytokines like TNF-α, IL-6, and IL-1β in the brain tissues of MCAO rats. This intervention also boosts HIF-1α expression while inhibiting the upregulation of ACSL4, thereby reducing ferroptosis and aiding in the recovery of function following brain I/R injury (156).

Exosomes are tiny membrane-bound vesicles that are expelled from cells and are being researched for their potential in treating a range of neurological conditions (157). In cases of cerebral ischemia, exosomes have demonstrated their ability to lessen neuronal injury and enhance the brain’s microenvironment by modulating inflammation, preventing ferroptosis, mediating apoptosis in cells, fostering axonal development, and facilitating vascular remodeling (158). Research by Wang et al. revealed that (159) exosomes derived from adipose stem cells (ADSC-Exo) act as inhibitors of ferroptosis, significantly decreasing M2 microglial sensitivity to this process and suppressing M1 microglial marker expression through the Fxr2/Atf3/Slc7a11 signaling pathway in MCAO mice. Furthermore, these exosomes diminish the levels of MDA, ROS, and Fe2^+^, leading to a decrease in pro-inflammatory cytokines such as IL-1β, IL-2, IL-6, IL-12 p70, IL-23, TNF-α, while simultaneously increasing the levels of anti-inflammatory cytokines like IL-10, TGF-β1, IL-4, and IL-13, which further mitigates inflammation and supports neuronal survival. Additionally, exosomes that overexpress steroid receptor coactivator-3 (SRC-3-exo) significantly elevate the GSH/GSSG ratio while decreasing the activity of catalase, MDA, and LPO, thereby preventing neuronal ferroptosis induced by OGD/R. This action also leads to the inhibition of microglial and astrocytic activation, resulting in a lowered production of pro-inflammatory cytokines such as IL-1β, IL-6, and TNF-α in the brains of MCAO mice. SRC3-exo treatment additionally reduces brain edema and infarction size in mice, promoting neuronal recovery and enhancing neurological performance (160). These findings suggest that exosomes may bolster the antioxidant defense mechanisms to counteract LPO-induced ferroptosis during cerebral I/R, further inhibiting the overactivation of neuroglial cells that plays a role in neuroinflammation. Additionally, extracellular vesicles derived from bone marrow mesenchymal stem cells (MSCs-EVs) are capable of transporting Fer-1 to neurons in the hippocampus. The combination of MSCs-EVs and Fer-1 enhances the levels of GPX4, suppresses COX-2 expression, and decreases the expression of TNF-α and IL-1β via the COX-2/PGE2 signaling pathway, thereby reducing neuroinflammation caused by ferroptosis and offering protection during cerebral I/R events (161).

### 5.4 Clinical feasibility and potential risks of treatment

The treatment strategies derived from basic research provide promising avenues for addressing the interplay between ferroptosis and inflammation in brain I/R. Anti-inflammatory drugs and environmental enrichment (EE) reduce inflammation by regulating pathways like HMGB1, Nrf2/NF-κB, TLR/p38 MAPK, and IL-6/STAT3, thereby mitigating ferroptosis (91, 124, 131, 155). Ferroptosis inhibitors and exosomes, on the other hand, target COX-2/PGE2, 5-LOX/leukotrienes, and Nrf2-mediated pathways, reducing the inflammatory impact of ferroptosis on inflammation (147, 152, 161). Innovations such as the regulation of M1/M2 microglial polarization by NTF, which influences the BMP6/SMADs pathway, demonstrate novel mechanisms to alleviate inflammation-induced ferroptosis (86). Bone morphogenetic protein 6 (BMP6), a regulator of hepcidin and iron metabolism, is upregulated in response to increased iron levels, triggering a SMAD signaling cascade that activates the hepcidin gene—a potential new approach to manage iron level alterations caused by microglia (162). Additionally, the ferroptosis inhibitor ADSC-Exo protects M2 microglia through the Fxr2/Atf3/Slc7a11 pathway, managing the inflammatory microenvironment (163, 164). Under ADSC-Exo’s influence, Fxr2 is upregulated, indirectly suppressing Atf3 while boosting SLC7A11 under oxidative stress, thus enhancing antioxidant defenses and inhibiting ferroptosis (159). New pharmacological developments like ADA-409-052 target the COX-2/PGE2 pathway to improve outcomes in severe neuronal death and neuroinflammation (142), while AA9 reduces hippocampal damage, inhibits iron deposition and ROS accumulation to alleviate ferroptosis, and subsequently suppresses neuroinflammation via the Nrf2 pathways (152).

Despite their innovative mechanisms offering new therapeutic possibilities, the potential risks and side effects of these strategies cannot be ignored. When evaluating their large-scale clinical application, initial small-scale clinical trials are crucial. These trials should thoroughly assess patient-specific conditions, drug safety, and potential interactions with other treatments. Possible side effects include immune suppression and elevated blood pressure from anti-inflammatory drugs. For instance, glycyrrhizin might increase blood pressure and lead to hypokalemia and hypernatremia—risks that necessitate cautious use, especially in hypertensive patients (165). Dimethyl fumarate may cause lymphocytopenia, a risk manageable with preemptive blood tests (166). Furthermore, using high doses of ferroptosis inhibitors, like caffeic acid, might impair iron absorption, leading to deficiency if not carefully managed (167).

Additionally, treatment plans should be tailored based on patient age, gender, and the specific brain regions affected. For example, elderly patients, who typically have diminished neuroplasticity and are more susceptible to iron accumulation, might benefit from combinations of iron chelators and stem cell exosomes (168). Female patients, potentially responding better to treatments due to the neuroprotective effects of estrogen, may have better outcomes (169, 170). Regional differences in brain sensitivity also dictate tailored approaches; for instance, the hippocampal region’s high demand for oxygen and glucose and its active iron metabolism make it particularly vulnerable to ischemic damage and ferroptosis. Therefore, treatment of the hippocampal region may primarily involve ferroptosis inhibitors, coupled with appropriate anti-inflammatory agents, and improving mitochondrial energy supply to neurons to support their survival and functional recovery (171, 172). The basal ganglia, critical for controlling muscle strength and tone, are frequently affected by strokes (173). Cerebral ischemia/reperfusion can increase the permeability of the blood-brain barrier (BBB) in this region (174). Therefore, we should increase the use of anti-inflammatory drugs and employ drug delivery systems to more effectively target the basal ganglia, protecting the integrity of the BBB. Additionally, combining ferroptosis inhibitors and motor neuron nutrients should be considered to mitigate the effects of ferroptosis and inflammation in this area. By fully understanding these variables, clinicians can develop more effective, personalized treatment strategies, ultimately improving patient outcomes in brain I/R scenarios.

## 6 Conclusion and prospects

Ferroptosis, an iron-dependent form of cell death, has garnered increasing attention for its role in ischemic brain diseases in recent years. Current research indicates that during ischemia-reperfusion injury, the accumulation of iron ions promotes lipid peroxidation by catalyzing ROS, a critical mechanism underlying ferroptosis. Furthermore, the inflammatory response significantly contributes to cerebral I/R injury. The activation of microglial cells leads to the release of numerous inflammatory mediators, which further exacerbate brain tissue damage. Ferroptosis not only induces direct neuronal death but may also worsen cerebral I/R injury by activating neuroglial cells and associated pathways that release inflammatory factors. While inflammation serves as a self-defense mechanism, excessive inflammation can be detrimental, resulting in iron accumulation and LPO-induced ferroptosis.

From a therapeutic standpoint, novel iron chelators and anti-inflammatory drugs have shown promise in experimental studies. For instance, the Ferrostatin-1 analog Srs11-92 has been demonstrated to reduce iron accumulation and activate the Nrf2 pathway, thereby alleviating both ferroptosis and inflammatory responses (152). Additionally, caffeic acid has been found to mitigate inflammation by inhibiting COX-2 and 5-LOX-induced inflammatory LPO (139, 141). Targeted treatment of inflammatory pathways, such as the administration of Salvia miltiorrhiza, significantly inhibits the activation of neuroglial cells, downregulates the expression of TNF-α, IL-6, and the phosphorylation of STAT3, thereby suppressing neuroinflammation-induced ferroptosis (135). Similarly, glycyrrhizin inhibits neuroinflammation-induced neuronal ferroptosis by downregulating TNF-α, IL-6, and IL-1β expression through the HMGB1/GPX4 pathway, demonstrating protective effects against ischemic brain damage (124).

Despite recent advances, the crosstalk mechanisms between ferroptosis and inflammation in cerebral I/R injury remain incompletely understood. Future research must delve deeper into the interaction mechanisms between these two processes and identify more effective strategies for their regulation to optimize therapeutic outcomes. For instance, while ferroptosis inhibitors have been shown to alleviate both ferroptosis and inflammation, the specific interaction mechanisms between them are not fully elucidated, and the potential health risks associated with their long-term use remain uncertain. Similarly, although inhibiting ACSL4 can mitigate both ferroptosis and inflammation, the upstream and downstream factors governing ACSL4 expression and activity, as well as their interactions under varying physiological and pathological conditions, are not yet fully understood, highlighting the need for further foundational research. Additionally, clinical studies are essential to validate the translatability and safety of findings from laboratory research. Future research directions should include the development of novel iron chelators and anti-inflammatory agents, alongside the exploration of combination therapy strategies. Moreover, advancements in biomarkers and imaging techniques will facilitate the early identification and quantitative assessment of the extent of ferroptosis and inflammation, thereby providing more precise timing and targets for clinical interventions. Through these comprehensive strategies, we aspire to achieve more effective treatment and prevention of cerebral I/R in the future.

In summary, the interplay between ferroptosis and inflammation in cerebral I/R injury represents a complex yet promising field of investigation. By thoroughly examining the interaction and regulatory mechanisms of these two processes, we can enhance our understanding of cerebral I/R development and establish a theoretical foundation for the development of novel therapeutic approaches.
